# Antibiotic stewardship bundle for uncomplicated gram-negative bacteremia at an academic health system: a quasi-experimental study

**DOI:** 10.1017/ash.2024.395

**Published:** 2024-10-10

**Authors:** Juliana DiPietro, Yanina Dubrovskaya, Kassandra Marsh, Arnold Decano, John Papadopoulos, Dana Mazo, Kenneth Inglima, Vincent Major, Jonathon So, Samuel Yuditskiy, Justin Siegfried

**Affiliations:** 1 Department of Pharmacy, NYU Langone Health, New York, NY, USA; 2 Division of Infectious Diseases, Department of Medicine, NYU Langone Health, New York, NY, USA; 3 Grossman School of Medicine, New York University, New York, NY, USA; 4 Department of Pathology, Grossman School of Medicine, New York University, New York, NY, USA; 5 Department of Population Health, NYU Langone Health, New York, NY, USA

## Abstract

**Objective:**

To evaluate whether an antimicrobial stewardship bundle (ASB) can safely empower frontline providers in the treatment of gram-negative bloodstream infections (GN-BSI).

**Intervention and Method:**

From March 2021 to February 2022, we implemented an ASB intervention for GN-BSI in the electronic medical record (EMR) to guide clinicians at the point of care to optimize their own antibiotic decision-making. We conducted a before-and-after quasi-experimental pre-bundle (preBG) and post-bundle (postBG) study evaluating a composite of in-hospital mortality, infection-related readmission, GN-BSI recurrence, and bundle-related outcomes.

**Setting:**

New York University Langone Health (NYULH), Tisch/Kimmel (T/K) and Brooklyn (BK) campuses, in New York City, New York.

**Patients:**

Out of 1097 patients screened, the study included 225 adults aged ≥18 years (101 preBG vs 124 postBG) admitted with at least one positive blood culture with a monomicrobial gram-negative organism.

**Results:**

There was no difference in the primary composite outcome (12.9% preBG vs. 7.3% postBG; *P* = 0.159) nor its individual components of in-hospital mortality, 30-day infection-related readmission, and GN-BSI recurrence. Vancomycin (VAN) discontinuation (DC) was done more frequently by the primary team in postBG (37.9% vs 66.7%; *P* < 0.001). In postBG, de-escalation done by the primary team increased by 8.8%, *P* = 0.310 and there was an 11.1% increase in the use of aminopenicillin-based antibiotics, *P* = 0.043.

**Conclusions:**

GN-BSI bundle worked as a nudge-based strategy to guide providers in VAN DC and increased de-escalation to aminopenicillin-based antibiotics without negatively impacting patient outcomes.

## Introduction

GN-BSI remain an important healthcare threat and one of the leading causes of both community and nosocomial-onset bacteremia worldwide.^1^ GN-BSI is also associated with significant mortality ranging from 13% to 20% and optimal treatment strategies can impact patient outcomes.^2–5^

ASB provide concise steps to clinicians at the point of care to guide antibiotic decision-making and have been associated with improved patient outcomes.^6,7^ Studies have found ASB improved the use of empiric antibiotics, decreased the use of broad-spectrum antibiotics, and shortened time to de-escalation of antibiotics.^8–10^ The use of ASB is reported most commonly with *Staphylococcus aureus* BSI and there is limited evidence regarding GN-BSI bundles in conjunction with stewardship interventions. A retrospective single-center study found an ASB for GN-BSI was associated with reduced duration of therapy (14 days vs 10 days; p<0.001) and shorter time to oral switch (5 days vs 4 days; *P* = 0.046).^11^ Another single-center GN-BSI study found time to definitive therapy was significantly shorter after implementation of an ASB with rapid diagnostic testing (32.6 hours vs 10.5 hours; *P* < 0.001).^12^

Both studies required active ASP review and intervention, but do not evaluate the use of an ASB through nudging in microbiology reporting, which may be a promising strategy to influence antibiotic decision-making while empowering prescriber autonomy.^13^ By incorporating nudging recommendations directly in the microbiology susceptibility report, this may improve and/or guide antibiotic selection and duration of therapy. A review by Langford et al reports selective or cascade microbiology reporting as one of the most common nudging strategies, however there is a paucity of literature evaluating a framing approach, which utilizes comments and recommendations in a test result to guide antibiotic therapy.^13^ Additionally, the Infectious Diseases Society of America (IDSA) Guidelines for Antimicrobial Stewardship provide a weak recommendation regarding nudging strategies due to limited evidence evaluating this stewardship practice.^14^

At NYULH, our stewardship team conducts syndrome-specific prospective audit and feedback (PAF) prioritizing daily real-time blood culture review. In an effort to optimize treatment strategies, we implemented a GN-BSI bundle to provide evidence-based recommendations to frontline clinicians directly in the EMR. The purpose of this study is to evaluate the GN-BSI bundle on patient outcomes and examine how the implementation of a nudge-based strategy influences frontline provider antibiotic decision-making. We hypothesize the ASB will work as a nudge-based strategy to educate frontline providers in real-time and shift antibiotic decision-making ownership from stewardship to the primary team.

## Methods

### ASP at NYULH

The Antimicrobial Stewardship Program (ASP) at NYULH was established in 2008 and expanded to include three ID-trained clinical pharmacotherapy specialists (CPS), along with rounding CPS conducting bedside stewardship. At our institution, there are 10-14 rounding CPS in our intensive care, hematology/oncology, bone marrow transplant, solid organ transplant, and internal medicine units. The ID-trained CPS instruct and mentor rounding CPS, so that after an orientation period, the rounding CPS can conduct bedside stewardship, including antimicrobial recommendations, education, and multidisciplinary decision-making. Our ASP utilizes a combination of preauthorization for restricted antimicrobials and PAF as core intervention strategies. On weekdays, ID-trained pharmacists communicate recommendations daily (NYULH T/K: 8am-6pm, NYULH BK: 8am-4pm) directly to the primary team via the EMR or verbally via phone call and document all interventions in the EMR using an iVent system of pharmacy communication. In 2014, our ASP moved towards syndrome specific PAF including blood culture reviews 3 times daily at NYULH T/K and 2 times daily at NYULH BK.

### Study design and patient population

This was an institutional review board approved before-and-after quasi-experimental preBG and postBG study of adult patients admitted to NYULH T/K and NYULH BK hospitals with at least one positive blood culture growing a monomicrobial Gram-negative organism from 3/2019–2/2020 and 3/2021–2/2022. Only the first eligible encounter was included. We excluded patients with a hospital length of stay (HLOS) ≤72 hours, secondary gram-positive infection, and duration of therapy (DOT) ≥21 days for clinical reasons, such as source of bacteremia (i.e., osteomyelitis, endocarditis, meningitis) and/or documentation of lack of source control. Additionally, we excluded patients who died or enrolled in hospice ≤72 hours of positive blood culture (PBC) draw, who were not admitted to the hospital at the time of PBC draw, history of PBC with the same organism within the previous 90 days, and pregnant women. We also excluded patients admitted to a medical team with a rounding CPS in order to assess the bundle’s impact directly on the primary team since it would have been difficult to differentiate the effect of the bundle from bedside CPS recommendations. This study followed Strengthening the Reporting of Observation Studies in Epidemiology (STROBE) reporting guidelines.^15^

### Interventions

**Figure 1.**
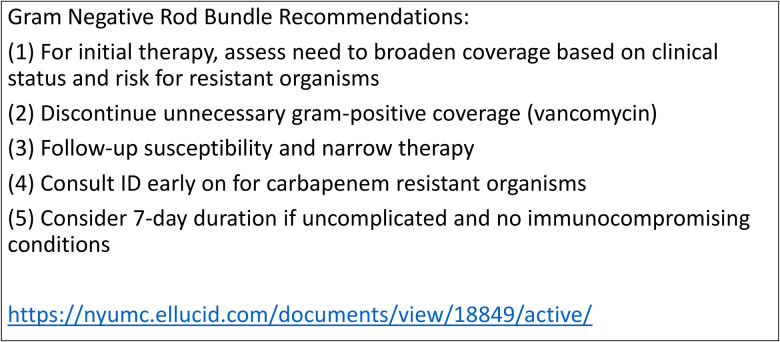
Gram-negative rod bacteremia bundle recommendations.

In March 2021, ASP and the microbiology lab incorporated a nudge-based bundle of evidence-based recommendations in the EMR microbiology report of a Gram-negative blood culture (Figure 1). The bundle populates initially when the blood culture growth is detected and repeats with each microbiology lab update. The bundle also includes a link to our institutional GN-BSI guidelines, which provides clinicians with information regarding the microbiology’s workflow, empiric therapy recommendations, de-escalation strategies, guidance on duration of therapy and references supporting the guidelines (Supplementary Material). The preBG represents patients with GN-BSI prior to March 2021, when the ASP team conducted blood culture PAF, and the postBG represents patients with GN-BSI after March 2021, which aligns with the implementation of the bundle in the EMR, in addition to the ASP blood culture PAF.

### Outcomes

The primary outcome was the composite of in-hospital mortality, infection-related readmission within 30 days, and GN-BSI recurrence within 30 days. Other patient-related outcomes included HLOS after PBC, total duration of antimicrobial therapy, and *Clostridioides difficile* (*C. difficile*) infection within 30 days. Bundle-related outcomes included the total duration of vancomycin therapy, vancomycin discontinuing team, de-escalation in patients who were candidates, time from intravenous (IV) to oral (PO) switch, percent of patients with ≤8 days for duration of therapy (DOT), and time from PBC to infectious diseases (ID) consultation for carbapenem-resistant *Enterobacterales* (CRE) organisms.

Empiric therapy was defined as an antimicrobial agent that was administered for the greatest portion of the first 48 hours from date of culture collection. Targeted therapy was defined as an antimicrobial agent with *in vitro* activity against the definitive organism growing on the blood culture that was administered for the greatest portion of time between 48 hours after culture collection and the end of inpatient treatment. Broad spectrum agents included piperacillin/tazobactam, 4^th^ generation cephalosporins or higher, carbapenems, and fluoroquinolones. Narrow-spectrum agents included aminopenicillins, 2^nd^ and 3^rd^ generation cephalosporins, and sulfamethoxazole/trimethoprim. Total DOT was defined as the difference in time between the initiation of the first IV antimicrobial agent and end of therapy. Time from IV to PO switch was defined as the difference in time between the initiation of the first IV antimicrobial agent and the initiation of a PO agent with *in vitro* activity to the infecting organism.

Eligible patients were identified from a microbiology laboratory report and by review of the EMR. Structured data (Charlson Comorbidity Index^(8)^ and Modified Pitt Bacteremia Score) were extracted from the EMR utilizing ICD-9/-10 codes and 10% of the total sample was manually verified for accuracy. All other data was manually collected from the EMR.

### Statistical analysis

Data was analyzed utilizing SPSS version 28.0.1.1 (IBM Corp, Armonk, New York). Categorical data was analyzed utilizing Pearson chi-square and Fisher’s exact test. Non-parametric data was analyzed utilizing Mann-Whitney *U* test. Statistical significance was defined by a 2-sided *P* value of <0.05.

## Results

### Patient characteristics

Of the 1097 patients with GN-BSI, 225 met inclusion criteria, with 101 patients in preBG and 124 patients in postBG. The most common reason for exclusion in both groups was having a rounding CPS on the primary team, followed by LOS ≤72 hours and secondary gram-positive infection (Supplementary Figure 1). The median (IQR) age was 81 years (70–90) in preBG and 76 years (61–84) in postBG (*P* = 0.056). Baseline characteristics were similar between groups (Table 1). In postBG, there were significantly more patients with diabetes mellitus (30.7% vs 45.2%; *P* = 0.027), renal disease (5% vs 44.4%; *P* < 0.001), and hemodialysis (0 vs 4.8%; *P* = 0.034). The preBG had more patients with moderate to severe liver disease (16.8% vs 4.8%; *P* = 0.003). The median (IQR) Charlson Comorbidity Index and Modified PITT Bacteremia Score were similar in both groups [4 (3–6) preBG vs 4 (2–5) postBG; *P* = 0.383 and 1 (0–2) preBG vs 1 (0–2) postBG; *P* = 0.797].

**Table 1.** Baseline and bacteremia characteristics

	Total n = 225	Pre-BG n = 101	Post-BG n = 124	***P*** **value**
**Patient characteristics**				
Institution, no. (%)				
Tisch/Kimmel Hospital	111 (49.3)	50 (49.5)	61 (49.2)	0.963
Brooklyn Hospital	114 (50.7)	51 (50.5)	63 (50.8)	
Age, years, median (IQR)	78 (66–86.5)	81 (70–90)	76 (61–84)	0.056
Male, no. (%)	116 (51.6)	48 (47.5)	68 (54.8)	0.275
Actual weight, kg, median (IQR)	70.2 (57.1–84.6)	71.7 (58–84.2)	70.2 (56.7–84.9)	0.952
BMI, kg/m^2^, median, (IQR)	25.3 (21.2–29.2)	25.3 (21.1–29)	25.4 (21.5–29.3)	0.952
Mechanical Ventilation, no. (%)	3 (1.3)	1 (1)	2 (1.6)	1
**Comorbidities**				
Diabetes mellitus, no. (%)	87 (38.7)	31 (30.7)	56 (45.2)	0.027
Renal Disease, no. (%)	60 (26.7)	5 (5)	55 (44.4)	< 0.001
Chronic Pulmonary Disease, no. (%)	57 (25.3)	25 (24.8)	32 (25.8)	0.857
Metastatic Solid Tumor, no. (%)	18 (8)	8 (7.9)	10 (8.1)	0.968
Moderate to Severe Liver Disease, no. (%)	23 (10.2)	17 (16.8)	6 (4.8)	0.003
Hemodialysis, no. (%)	6 (2.7)	0	6 (4.8)	0.034
Immunocompromised, no. (%)	45 (20)	22 (21.8)	23 (18.5)	0.546
Leukemia	4 (1.8)	0	4 (3.2)	0.130
Lymphoma	7 (3.1)	4 (4)	3 (2.4)	0.703
HIV/AIDS	16 (7.1)	14 (13.9)	2 (1.6)	< 0.001
Immunosuppressive Medication	27 (12)	9 (8.9)	18 (14.5)	0.198
Charlson Comorbidity Index, median (IQR)	4 (2–5)	4 (3–6)	4 (2–5)	0.383
Modified PITT Bacteremia Score, median (IQR)	1 (1–2)	1 (0–2)	1 (0–2)	0.797
**Bacteremia Characteristics**				
Source of bacteremia, no. (%)				
Urinary	133 (59.1)	60 (59.4)	73 (58.9)	0.935
Intra-abdominal	58 (25.8)	29 (28.7)	29 (23.4)	0.364
Respiratory	17 (7.6)	7 (6.9)	10 (8.1)	0.749
Catheter-Related	8 (3.6)	0	8 (6.5)	0.009
SSTI	5 (2.2)	3 (3)	2 (1.6)	0.659
Unknown	4 (1.8)	2 (2)	2 (1.6)	1
Organism, no. (%)				
*Escherichia coli*	138 (61.3)	63 (62.4)	75 (60.5)	0.772
*Klebsiella* spp.	46 (20.4)	20 (29.8)	26 (21)	0.829
*Proteus* spp.	16 (7.1)	5 (5)	11 (8.9)	0.255
Other^1^	25 (11.1)	13 (12.9)	12 (9.7)	0.448
Susceptibility Data, no. (%)				
ESBL	48 (21.3)	20 (19.8)	28 (22.6)	0.613
CRE	2 (0.9)	1 (1)	1 (0.8)	1
Ampicillin/Sulbactam Resistance	77/202 (38.1)	35/86 (40.7)	42/116 (36.2)	0.516

IQR, interquartile range; ESBL, extended-spectrum beta -lactamase; CRE, carbapenem-resistant Enterobacterales.

1 Other: *Serratia marcescens*, *Pseudomonas* spp., *Citrobacter* spp., *Enterobacter cloacae*, and *Morganella morganii*.

### Bacteremia characteristics

In both groups, urine was the most common source of bacteremia (59.4% preBG vs 58.9% postBG; *P* = 0.935), followed by intra-abdominal (28.7% preBG vs 23.4% postBG; *P* = 0.364). *Escherichia coli* was the most commonly isolated organism (62.4% preBG vs 60.5% postBG; *P* = 0.772), followed by *Klebsiella* species (29.8% preBG vs 21% postBG; *P* = 0.829). Rates of ESBL and CRE were similar in both groups. Bacteremia characteristics can be found in Table 1.

### Treatment characteristics

Empiric antibiotic use was similar between the two groups. The most common empiric antibiotic was piperacillin/tazobactam (80.2% preBG vs 76.8% postBG; *P* = 0.584). The percentage of patients who received inactive empiric therapy was similar (5% preBG vs 12.1% postBG; *P* = 0.061). Narrow-spectrum antimicrobial agents were used as targeted therapy in 52.3% patients in preBG and in 54% patients in postBG (*P* = 0.816). Significantly more patients in postBG were narrowed to ampicillin/sulbactam or amoxicillin/clavulanate (3.8% vs 14.9%; *P* = 0.043). IV to PO switch and time to switch while inpatient was similar in both groups. Treatment characteristics can be found in Table 2.

**Table 2.** Treatment characteristics

	Total	Pre-BG	Post-BG	***P*** **Value**
**Targeted Therapy, no. (%)**				
Narrow Spectrum	120 (53.3)	53 (52.3)	67 (54)	0.816
3^rd^ generation cephalosporin	100/120 (83.3)	46/53 (86.8)	54/67 (80.6)	0.366
Ampicillin/sulbactam or amoxicillin/clavulanate	12/120 (10)	2/53 (3.8)	10/67 (14.9)	0.043
Broad Spectrum	105 (46.7)	48 (47.5)	57 (46)	0.816
Piperacillin/tazobactam	54/105 (51.4)	27/48 (56.3)	27/57 (47.4)	0.364
Carbapenems	34/105 (32.4)	13/48 (27.1)	21/57 (36.8)	0.287
Fluoroquinolones	8/105 (7.6)	5/48 (10.4)	3/57 (5.3)	0.456
**Discharge Antibiotic Therapy, no. (%)**	190 (84.4)	80 (79.2)	110 (88.7)	0.050
Narrow Spectrum	100/190 (52.6)	42/80 (52.5)	58/110 (52.7)	0.975
Cefuroxime	10/100 (10)	3/42 (7.1)	7/58 (12.1)	0.513
3^rd^ generation cephalosporin	50/100 (50)	23/42 (54.8)	27/58 (46.6)	0.418
Ampicillin/sulbactam or amoxicillin/clavulanate	27/100 (27)	9/42 (21.4)	18/58 (31)	0.286
Trimethoprim/sulfamethoxazole	11/100 (11)	5/42 (11.9)	6/58 (10.3)	0.806
Broad Spectrum	90/100 (47.4)	38/80 (47.5)	52/110 (47.3)	0.975
Fluoroquinolones	63/90 (70)	31/38 (81.6)	32/52 (61.5)	0.040
Carbapenems	24/90 (26.7)	7/38 (18.4)	17/52 (32.7)	0.130
IV to PO switch while inpatient, no. (%)	47 (20.9)	26 (25.7)	21 (16.9)	0.106
Time from IV to PO switch, days, median (IQR)	5 (4–6)	5 (4–7)	5 (4–5)	0.146
Candidate for De-escalation	150 (66.7)	75 (74.3)	75 (60.5)	0.029

### Outcomes

The primary composite outcome of in-hospital mortality, infection-related readmission within 30 days of discharge, and GN-BSI recurrence within 30 days of discharge was similar (12.9% preBG vs 7.3% postBG; *P* = 0.159) (Table 3). For secondary outcomes, median (IQR) HLOS from onset of bacteremia was 5 days (3.5–5) in preBG and 4.7 days (3–7) in postBG (*P* = 0.619). Median (IQR) DOT and *C. difficile* infection was similar [14 days (12–15) preBG vs 15 days (11.25–16) postBG; *P* = 0.277 and (3%) preBG vs (1.6%) postBG; *P* = 0.659] (Table 3).

**Table 3.** Primary and secondary outcomes

	Total	Pre-Intervention	Post-Intervention	***P*** **Value**
Primary composite outcome, no. (%)	22 (9.8)	13 (12.9)	9 (7.3)	0.159
In-hospital mortality	2 (0.9)	2 (2)	0	0.2
Any infection-related readmission within 30 days of discharge	19 (8.4)	10 (9.9)	9 (7.3)	0.478
GN-BSI Recurrence within 30 days	3 (1.3)	1 (1)	2 (1.6)	1
Secondary outcomes, no (%)				
Hospital LOS from onset of bacteremia, days, median (IQR)	5 (3–7)	5 (3.5–5)	4.7 (3–7)	0.619
Total duration of antimicrobial therapy, days, median (IQR)	15 (12-16)	14 (12–15)	15 (11.25–16)	0.277
*C. difficile* infection within 30 days of bacterial growth detection, no. (%)	5 (2.2)	3 (3)	2 (1.6)	0.659

### Bundle-related outcomes

Vancomycin was empirically used in 57.4% of patients in preBG and in 60.5% in postBG (*P* = 0.643). The total duration of vancomycin therapy was a median (IQR) of 2 days (1–2) in preBG compared to 2 days (1–2) in postBG (*P* = 0.718). Vancomycin was discontinued significantly more by the primary team in postBG (37.9% vs 66.7%; *P* < 0.001).

There were numerically more patients who were de-escalated in postBG (73.3% vs 81.3%; *P* = 0.242) with an 8.8% increase in de-escalation done by the primary team in postBG (*P* = 0.310). There was no difference in the number of patients who received <8 days of therapy (7% preBG vs 3% postBG; *P* = 0.594). The rate of ID consultations was similar (67.3% preBG vs 56.5% postBG; *P* = 0.096). The median time (IQR) from bacterial growth to ID consultation was significantly longer in postBG [1.4 days (0.5–1.9) vs 1.7 days (1.2–2.7); *P* = 0.011]. Of interest, ID consultation for ESBL-producing organisms was significantly decreased in postBG (90% vs 64.3%; *P* = 0.043). There were only 2 cases of CRE and both cases had ID consultation. A full summary of bundle-related outcomes can be found in supplementary table 1.

## Discussion

Overall, we found adding GN-BSI bundle recommendations in the EMR was successful without negatively impacting patient outcomes. There was a statistically significant increase in the primary team’s ownership of vancomycin discontinuation (37.9% vs 66.7%; *P* < 0.001) and a numerical increase in antimicrobial de-escalation (27.3% vs 36.1%; *P* = 0.310). Also, significantly more patients were narrowed to aminopenicillins in postBG (3.8% vs 14.9%; *P* = 0.043). Our study results are similar to previous experience. Musgrove et al reported using a simple microbiology comment nudge improved pneumonia prescribing practices. In their study, respiratory cultures with no dominant organism growth were reported by the clinical microbiology laboratory as “commensal respiratory flora only: No *S. aureus*/MRSA [methicillin-resistant *Staphylococcus aureus*] or *P.* [*Pseudomonas*] *aeruginosa.”* They found de-escalation/discontinuation of unnecessary broad-spectrum antibiotics was more commonly performed in the intervention group without causing harm (39% vs 73%, *P* < .001).^16^

Erickson et al showed a decrease in DOT (14 vs 10 days; *P* < 0.01) and a decrease in time to IV to PO switch (5 days vs. 4 days; *P* = 0.46), while Walsh et al showed a decrease in time to definitive therapy (32.6 hours vs 10.5 hours; *P* < 0.001).^11,12^ There were no differences in these outcomes in our study. In Erickson and Walsh, ASP PAF and rapid diagnostic testing were part of the bundles under evaluation, while these interventions were constant across our study’s groups: ASP PAF occurred in both time periods and rapid diagnostics in neither. In addition, the main goal of our GN-BSI bundle was to utilize the bundle as a passive nudge-based strategy to empower the primary team to discontinue unnecessary empiric vancomycin and perform antimicrobial de-escalation on their own without ASP intervention.

The nudge-based recommendation to discontinue unnecessary vancomycin led to a shift where the primary team stopped vancomycin without prompting from either ASP or ID consultation. PAF can often be labor intensive and nudge-based bundle recommendations provided to the clinician at the point of care can facilitate optimal therapy by stopping unnecessary empiric broad-spectrum antimicrobials.^13,14^ There also was a numerical increase in de-escalation done by the primary team and an increase in the use of narrow spectrum agents with aminopenicillin therapy based on the bundle recommendations.

The order and passive nature of the bundle recommendations may have affected the impact of each individual guidance, with later recommendations receiving less consideration. Discontinuation of vancomycin was the 2^nd^ recommendation in the bundle and the intervention primary teams followed most often on their own, while there was no change seen in the last recommendation to consider a 7-day DOT. In addition, we hypothesize that the lack of change between the groups in de-escalation done by the primary team may result from the need for a higher level of ID training and comfortability in interpreting susceptibility data when selecting targeted narrow-spectrum therapy.

In postBG, we found a slightly longer time to ID consultation resulting in a median of 7.2 hours later. There was also a trend towards decreased ID consultation (*P* = 0.096) which reached statistical significance for patients growing ESBL-producing organisms (90% preBG vs 64.3% postBG; *P* = 0.043). These finding may be attributed to the primary team’s increased comfort with the initial treatment of GN-BSI facilitated by the bundle, which included a reference link to our institutional treatment guidelines. The decrease in ID consultation for ESBL-producing organisms may also have been a result of the bundle recommending ID consultation specifically for CRE. This delay and decreased ID consultation for ESBLs without findings of any impact on clinical outcomes, suggests the bundle may allow for improved use of ID consultation resources, by decreasing unnecessary consults.

The last recommendation in the bundle is to consider a 7-day DOT for uncomplicated GN-BSI in patients with no immunocompromising conditions. Our study showed the bundle did not influence antimicrobial DOT. When we compare the DOT seen in the included population to the population excluded due to rounding CPS on the primary team, 7-day DOT still remained low (in excluded patients: 6% preBG vs 15% postBG, *P* = 0.112). A possible contributing factor to this finding includes the placement of the 7-day DOT as the last recommendation of the bundle. In addition, considering urine was the most common source of bacteremia, our institutional guidelines for urinary tract infections at the time may have influenced this result, as these guidelines recommend 7–14 days for complicated cystitis and 10–14 days for pyelonephritis. Primary teams may have used those guidelines defaulting to the longer durations over recommendations in the GN-BSI guidelines.

Our study had several limitations. First, this was a before-and-after, quasi-experimental study design and cannot account for confounding factors or changes in practice over time compared to a randomized study. Although the bundle populates and remains visible in the EMR, we cannot confirm the primary team fully utilized or read the entirety of the GN-BSI bundled recommendations. It is possible that the changes in postBG could be caused by other variables other than the bundle. Second, we excluded patients in the ICU or who are immunocompromised, which limits the generalizability in these specific populations. Third, the primary composite outcome only evaluated readmission or infection recurrences within our institution and did not account for admissions or recurrences from outside hospitals. Fourth, by including 2^nd^ and 3^rd^ generation cephalosporins in our definition of narrow spectrum therapy, this may not be consistent with other antibiotic use classifications. However, this is a common de-escalation practice at our institution since our automated susceptibility testing platform, at the study time, did not measure cefazolin MICs to below the breakpoint for bacteremia.

Overall, the use of a nudge-based GN-BSI bundle in the EMR showed an increase in vancomycin discontinuation by the primary team and a trend towards an increase in antimicrobial de-escalation without negatively affecting patient specific outcomes. This nudge-based bundle may be a useful strategy as it empowers the primary team to have more ownership in their antimicrobial decision-making and can allow ASP and ID consultants to focus their time on higher-level interventions. Additional research should evaluate the use of different and/or a combination nudging strategies to identify the most effective approach to this stewardship practice as well as the impact of this concept to less resourced programs.

## Supporting information

DiPietro et al. supplementary material DiPietro et al. supplementary material

## Supplementary material

For supplementary material accompanying this paper visit http://doi.org/10.1017/ash.2024.395. click here to view supplementary material
