# Supplementary material for: Antibiotic stewardship bundle for uncomplicated gram-negative bacteremia at an academic health system: a quasi-experimental study
Source: Antimicrob Steward Healthc Epidemiol. 2024 Oct 10;4(1):e171. doi: 10.1017/ash.2024.395 (PMC11474889; doi:10.1017/ash.2024.395)
Supplement: DiPietro et al. supplementary material [file S2732494X24003954sup001.docx]

**Supplementary Appendix 1**

**Supplementary Institutional GN-BSI Bundle Guidelines:**

**CLINICAL CONTEXT AND PURPOSE:**

- To provide guidelines for the diagnosis and management of **Uncomplicated** **Gram-negative (GN)** bacteremia in adult patients
- **Recommendations are guidelines for care and are not meant to replace clinical judgment**
- Initial section includes key concepts for guidance with supporting evidence and algorithm describing management

**GN ANTIBIOTIC CARE BUNDLE:**

- Care bundles consist of a group of key evidence-based actions to provide a consistent strategy to ensure each patient receives the optimal and appropriate antibiotic treatment

**GN BUNDLE RECOMMENDATIONS:**

*See full treatment guideline for complete recommendations.*

1. For initial therapy, assess need to broaden coverage based on clinical status and risk for resistant organisms
2. Discontinue unnecessary gram-positive therapy (vancomycin)
3. Follow-up susceptibility and narrow therapy
4. Consider early ID consult for carbapenem-resistant organisms
5. Consider 7-day duration if uncomplicated and no immunocompromising conditions

**GUIDELINE RECOMMENDATIONS: KEY CONCEPTS**

1. **Microbiology Testing:**

| Testing Method | Microbiologic Information | Timing of result |
| --- | --- | --- |
| Routine gram stain | - Provides preliminary morphology of organism detected from positive blood culture | - Immediately following gram stain |
| Routine bacterial culture | - Provides final organism identification and complete panel of antimicrobial susceptibility results | - ~48 to 72 hours |

- **Microbiology Workflow:**

**Start or continue empiric antibiotics (see next section)**

**Final organism ID and susceptibility panel**

1. **Treatment phase – Empiric (initial) antimicrobial therapy selection:**

- **Suspected bloodstream infection**
- **Two sets of blood cultures obtained**
- **After cultures sent, empiric antibiotics initiated**

Blood culture(s) positive for

**Gram-negative rods**

**(Primary team notified by microbiology lab)**

**Yes**

- **Evaluate for and control primary source of infection**
- **Narrow antibiotic regimen based on final culture and susceptibility testing results once available (within 48 to 72 hours) and discontinue any unnecessary Gram-positive therapy (vancomycin)**

**Does the patient have sepsis or septic shock?***

**Meropenem +/-Amikacin (AG)***

**Sepsis/Septic Shock**

**Without Sepsis**

**Are any of the following present?**

- **History of MDRO colonization or infection**
- **Recent (<90 days) hospital/healthcare facility stay**
- **Currently hospitalized ≥5 days**
- **Recent broad spectrum antibiotic use**

**Are any of the following present?**

- **Recent (<90 days) hospital/healthcare facility stay or prior antibiotic use**
- **Immunocompromised/transplant patients**
- **Prior *Pseudomonas aeruginosa* infection in the prior 3 to 6 months**

**No**

**No**

**Yes**

**Piperacillin-tazobactam/ Cefepime***

**Ceftriaxone***

| **Definitions and alternative regimens due to penicillin allergy*** | |
| --- | --- |
| **Sepsis** | - Evidence of organ dysfunction, hypotensive (SBP <100), elevated lactate >2mmol/L |
| **Septic shock** | - Sepsis despite adequate fluid resuscitation, require vasopressors to maintain mean arterial pressure (MAP) ≥65 mmHg **(combination therapy with AG may be considered)** |
| **Beta-lactam allergy** | - Consult Allergy or ASP/ID |

1. **Treatment phase – Directed antimicrobial therapy selection: (Confirmed type of infection, causative pathogen, and antimicrobial susceptibilities)**
   - Once final culture results and antimicrobial susceptibility data are available, therapy should be tailored to the specific pathogen
     - If combination therapy was used empirically, the regimen should generally be switched to a single agent with the **narrowest spectrum** to which the organism is susceptible
     - **Early de-escalation (within ~48 to 72hr)** may preserve broad-spectrum agents, avoid selecting for resistant pathogens, and minimize risk of *Clostridioides difficile*
   - **Example of organism identification and susceptibility reporting in Epic:**


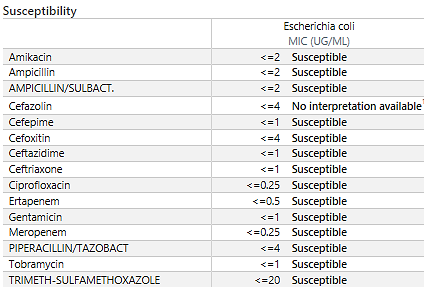


***If empirically started on piperacillin/tazobactam, consider de-escalation to narrowest therapy with ampicillin/sulbactam**

- - **If carbapenem-resistant organism isolated, strongly recommend to consult ID to assist with management as delay in time to appropriate therapy can significantly impact clinical outcomes (every day matters!)*****

1. **Route and duration of therapy:**
   - Initially, antibiotics should be given intravenously, but in select patients who have defervesced and remained **afebrile for 48 hours**, antibiotics may be switched to an oral agent with adequate bioavailability if the isolate is susceptible
     - Consider consultation with **ASP/clinical pharmacy/ID** for oral switch and duration recommendations once microbiology susceptibilities available
     - Streamlining to oral beta-lactams may be considered (sparing fluoroquinolone class)
   - Duration determined by clinical response in addition to primary source and extent of infection
     - In most cases, the duration of antibiotic therapy ranges from **7 to 14 days**
   - For uncomplicated infections with Enterobacterales, an antibiotic duration on the shorter end of the range above is as effective as a longer course and could reduce the selective pressure for antibiotic resistance and collateral damage (*Clostridioides difficile*)
     - Consider **7 days** for the following patient scenarios:

| **Duration of therapy** | **Parameters** |
| --- | --- |
| **7 days** | - Uncomplicated Enterobacterales bacteremia (e.g., *Escherichia coli, Klebsiella pneumonia, Proteus* spp.) - No underlying endovascular source, bone, joint or CNS infection - No uncontrolled source of infection (e.g., undrained abscess) - No major immunocompromising condition^1^ - Clinical improvement within 48 to 72 hours (afebrile, normotensive) |

^1^**Immunocompromising conditions** include B- or T-lymphocyte deficiency, complement deficiencies, non-dermatologic cancers without neutropenia, HIV with a CD4 cell count >200/mL^3^, chronic renal failure, nephrotic syndrome, chronic liver disease, radiation, active immunosuppressive therapy for other diseases (e.g., long-term steroid therapy with prednisone). Studies have shown that HIV with a CD4+ T cell count <200/mL^3^, severe neutropenia (ANC <100 cells/mm^3^) or presence of ≥2 conditions is warranted for definition of significantly immunocompromised

1. **Follow-up blood cultures:**

- For patients who clinically improve after the initiation of appropriate antibiotic therapy, repeat blood cultures to document clearance of bacteremia may be **unnecessary**
  - Persistent bacteremia, particularly when the source of infection has been controlled, is uncommon with Gram-negative pathogens
  - Repeating blood cultures may be warranted for patients who continue to be febrile or otherwise acutely ill (or relapse) despite antibiotic therapy or for those in whom source control has not been assured

**Supplementary Figure 1: Patient Selection**


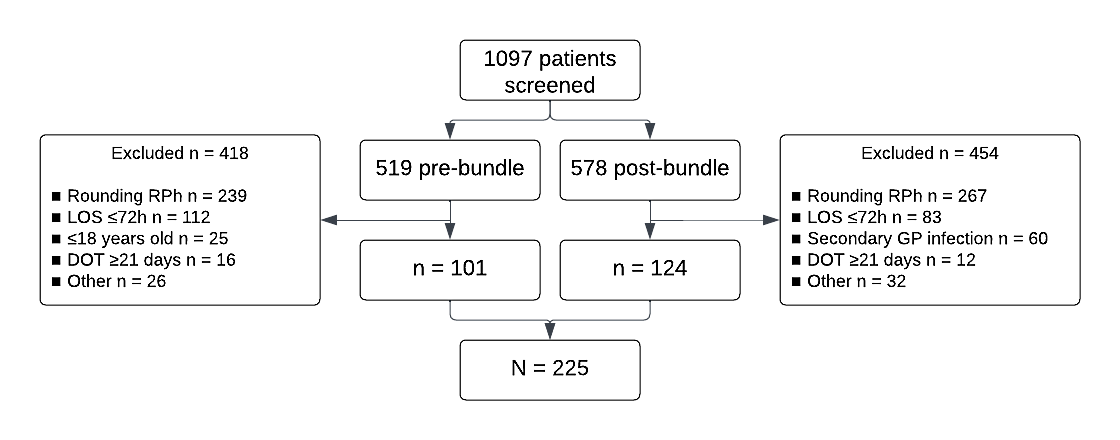


RPh: pharmacist

LOS: length of stay

GP: gram-positive

DOT: days of therapy

Other: fastidious organism, expiration of hospice ≤72 hours from positive blood culture draw, polymicrobial gram-negative blood stream infection

**Supplementary Table 1: Bundle-Related Outcomes**

|  | **Total** | **Pre-Intervention** | **Post-Intervention** | **P- Value** |
| --- | --- | --- | --- | --- |
| **Vancomycin-Bundle Related Outcomes** | | | | |
| Empiric Vancomycin | 133 (59.1) | 58 (57.4) | 75 (60.5) | 0.643 |
| Total duration of vancomycin therapy, days, median (IQR) | 2 (1 – 2) | 2 (1 – 2) | 2 (1 – 2) | 0.718 |
| Time from bacterial growth detection to discontinuation of vancomycin, days, median (IQR) | 0 (-1 – 1) | 0 (-1 – 1) | 0 (-1 – 0) | 0.135 |
| Vancomycin discontinuing team, no. (%) | | | | |
| Primary Team | 72/133 (54.1) | 22/58 (37.9) | 50/75 (66.7) | < 0.001 |
| ID Recommendation | 34/133 (25.6) | 21/58 (36.2) | 13/75 (17.3) | 0.013 |
| ASP Recommendation | 27/133 (20.3) | 15/58 (25.9) | 12/75 (16) | 0.161 |
| **De-escalation-Bundle Related Outcomes** | | | | |
| Time from culture susceptibilities reported to de-escalation (targeted therapy), days, median (IQR) | 0 (-2 – 0) | 0 (-2 – 0) | 0 (-2 – 1) | 0.440 |
| Candidate for De-escalation | 150 (66.7) | 75 (74.3) | 75 (60.5) | 0.029 |
| First de-escalation, no. (%) | 116/150 (77.3) | 55/75 (73.3) | 61/75 (81.3) | 0.242 |
| De-escalating team |  |  |  |  |
| Primary Team | 37/116 (31.9) | 15/55 (27.3) | 22/61 (36.1) | 0.310 |
| ID Recommendation | 36/116 (31) | 21/55 (38.2) | 15/61 (24.6) | 0.114 |
| ASP Recommendation | 43/116 (37.1) | 19/55 (34.9) | 24/61 (39.3) | 0.593 |
| Inpatient Escalation, no. (%) | 48 (21.3) | 22 (21.8) | 26 (21) | 0.882 |
| Appropriate escalation | 39/48 (81.3) | 18/22 (81.8) | 21/26 (80.8) | 1 |
| **Days of Therapy-Bundle Related Outcomes** | | | | |
| ≤ 8 days of antibiotic therapy, no. (%) | 18 (8) | 7 (7) | 11 (3) | 0.594 |
| **ID Consult-Bundle Related Outcomes** | | | | |
| ID consult, no. (%) | 138 (61.3) | 68 (67.3) | 70 (56.5) | 0.096 |
| Time from bacterial growth detection to ID consult, days, median (IQR) | 1.5 (0.6 – 2.5) | 1.4 (0.5 – 1.9) | 1.7 (1.2 – 2.7) | 0.011 |
| ID consult for ESBL | 36/48 (75) | 18/20 (90) | 18/28 (64.3) | 0.043 |
| ID consult for CRE | 2/2 (100) | 1/1 (100) | 1/1 (100) | - |
